# Non-invasive detection of instantaneous fetal hypoxemia in large animal model of pregnancy

**DOI:** 10.1038/s44385-025-00014-0

**Published:** 2025-04-23

**Authors:** Weitai Qian, Mahya Saffarpour, Rishad R. Joarder, Begum Kasap, Kourosh Vali, Tailai Lihe, Herman L. Hedriana, Aijun Wang, Diana Farmer, Soheil Ghiasi

**Affiliations:** 1 Department of Electrical and Computer Engineering, University of California, Davis, One Shields Avenue, Davis, CA 95616 USA; 2 Department of Obstetrics and Gynecology, University of California, Davis Health, 4301 X Street, Sacramento, CA 95817 USA; 3 Department of Surgery, University of California, Davis Health, 4301 X Street, Sacramento, CA 95817 USA; 4 Department of Biomedical Engineering, University of California, Davis, One Shields Avenue, Davis, CA 95616 USA

**Keywords:** Medical research, Preclinical research

## Abstract

Cardiotocography (CTG) has a high false positive rate for the detection of babies at risk of birth asphyxia. Transabdominal Fetal Pulse Oximetry (TFO) has the potential to supplement CTG by enabling non-invasive measurement of fetal arterial blood oxygen saturation (fSpO_2_). Previous attempts at TFO were limited to intermittent measurements using highly specialized and precise instruments. We present a TFO system, utilizing multiple commodity silicon photo-detectors to acquire mixed maternal-fetal PPG signals, for non-invasive and continuous detection of fetal instantaneous normoxia vs. hypoxemia status, relative to a user-specified threshold. Data from controlled de-saturation experiments using pregnant ewes with an in-utero hypoxic lamb model, from a total of *n* = 8 hypoxic rounds (length = 34.5 ± 12 min), is used to validate the technology. The multi-layer perceptron model is used for information fusion, and fetal arterial blood oxygen saturation obtained from blood gas analysis is used as a gold standard. The method detects instantaneous hypoxemia (fSpO_2_ <30%) with 87.6% accuracy. Cross-validation shows an average sensitivity of 88.2% and specificity of 71.2%. The receiver operating characteristic (ROC) curves showed strong discrimination abilities in all cross-validation iterations (AUC = 0.87). This study underscores TFO’s promise for accurate detection of instantaneous fetal hypoxemia relative to a user-defined threshold value, and for contribution to enhancement of intrapartum fetal monitoring in the longer term.

## Introduction

Electronic fetal monitoring (EFM), technically referred to as cardiotocography (CTG), is used for intrapartum fetal monitoring with the goal of early identification of fetal asphyxia, and preventing neonatal hypoxic-ischemic encephalopathy (HIE)^1^. Clinical trials were not conducted before the widespread adoption of EFM in the 1970s. Strikingly, the rate of Cesarean section (C-section) deliveries in the United States has increased about fivefold since then, while the rates of various conditions associated with HIE at birth, such as cerebral palsy, remain unchanged^2–6^.

The current paradigm for intrapartum EFM involves the interpretation of fetal heart rate (FHR) traces in the context of maternal uterine contractions, as a proxy for fetal hypoxic distress. The fetal hypoxic distress may lead to inadequate oxygen supply to the fetal brain cells and may cause HIE, thus interventions such as emergency C-section or operative vaginal delivery become compelling options^7–10^. EFM is highly sensitive for the detection of fetal hypoxia, i.e., virtually no significantly hypoxic baby will have a normal CTG tracing. The problem with CTG trace interpretation is that non-reassuring FHR traces may include fetuses both adapting normally, and compromised to hypoxia, while the difference between those two types of tracings could be inconsistently interpreted. That is, CTG is not specific enough, and has a high rate of false positives for the detection of babies at risk of birth asphyxia^11^.

**Fig. 1:**
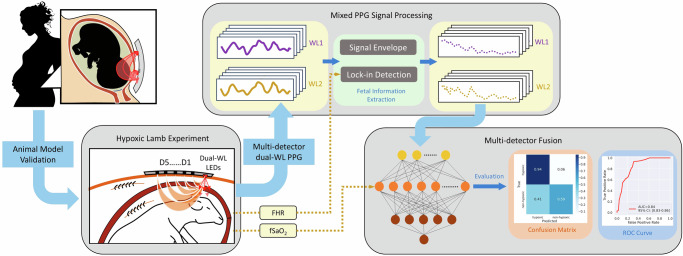
The pipeline of data collection and validation utilized in the paper. The results and methods for each step are discussed in the following sections.

Poor specificity of CTG for detection of babies at risk of birth asphyxia has been widely recognized^12,13^, and a number of alternative approaches for intrapartum fetal assessment, such as fetal ECG, biophysical profiling, and fetal scalp pH sampling, have been explored^14–18^. Although biochemical methods such as fetal scalp blood sampling provide high accuracy in quantification of fetal hypoxic distress, such methods have not proved effective for improving birth outcomes, in part due to their invasive mode, limited applicability to only after membrane rupture, and inability to support continuous fetal monitoring throughout labor and delivery. Aiming to enhance the set of tools available to obstetricians for intrapartum assessment of fetal hypoxic distress in the longer term, we developed the technology for non-invasive and continuous detection of instantaneous fetal hypoxemia, and present encouraging validation results in gold standard large animal model of pregnancy (Fig. 1).

Specifically, our team has developed a transabdominal fetal oximetry (TFO) device, which can collect dual-wavelength multi-detector photoplethysmogram (PPG) signals, from pregnant subjects^19,20^. The acquired PPG signals contain information from a mixture of maternal and fetal tissue layers, and are thus referred to as mixed-PPG signals. Mixed signals contain very faint contributions of the pulsatile fetal arteries to the signal.

TFO leverages the principles of pulse oximetry, rooted in the Beer–Lambert law (BLL), to non-invasively infer fetal arterial blood oxygen saturation from acquired mixed-PPG signals (fSpO_2_). Fundamentally, the sensing function relies on the change in the absorption spectra of fetal hemoglobin in its oxy- and deoxy- states in the far visible and near-infrared ranges of the electromagnetic spectrum. By analyzing the detected diffused light intensities at a minimum of two wavelengths and isolating fetal-induced changes from the mixed-PPG signals, the concentration ratio of oxygenated to total hemoglobin, referred to as oxygen saturation, is inferred relative to a given threshold. We develop and utilize techniques for processing the mixed-PPG signals to isolate the pulsatile changes in light absorption, which are caused by the fetal arteries in the field of sensing, and for detecting the instantaneous fSpO_2_ status, relative to a predefined target threshold.

Previous studies have highlighted the influence of change in fSpO_2_ on collected TFO signals, thereby demonstrating the conceptual feasibility of fSpO_2_ measurement^19,21^. The analysis, however, relied on extensive manual inspection, characterization, and tweaking of the data on a single subject, and reporting calibration (training) error obtained in very long averaging windows. As a result, continuous non-invasive measurement of fSpO_2_ remains an open problem in practice.

Aiming to contribute to the state of intrapartum fetal monitoring in the longer term, this paper demonstrates the feasibility of continuous non-invasive monitoring, and real-time detection of instantaneous fetal hypoxemia in preclinical models (Fig. 1). We envision that utilization of continuous TFO in conjunction with continuous EFM enables the development of novel subsequent studies on non-invasive assessment of fetal adaptation, and increased accuracy of detecting babies at risk of birth asphyxia.

## Results

Figure 1 sketches a high-level methodology utilized to obtain the raw data and results reported in this study. The pipeline is demonstrated as three steps including animal experiment overview, mixed-PPG processing, and detector fusion for fetal hypoxemia detection.

### Pregnant ewe with hypoxic in-utero fetal lamb model

**Fig. 2:**
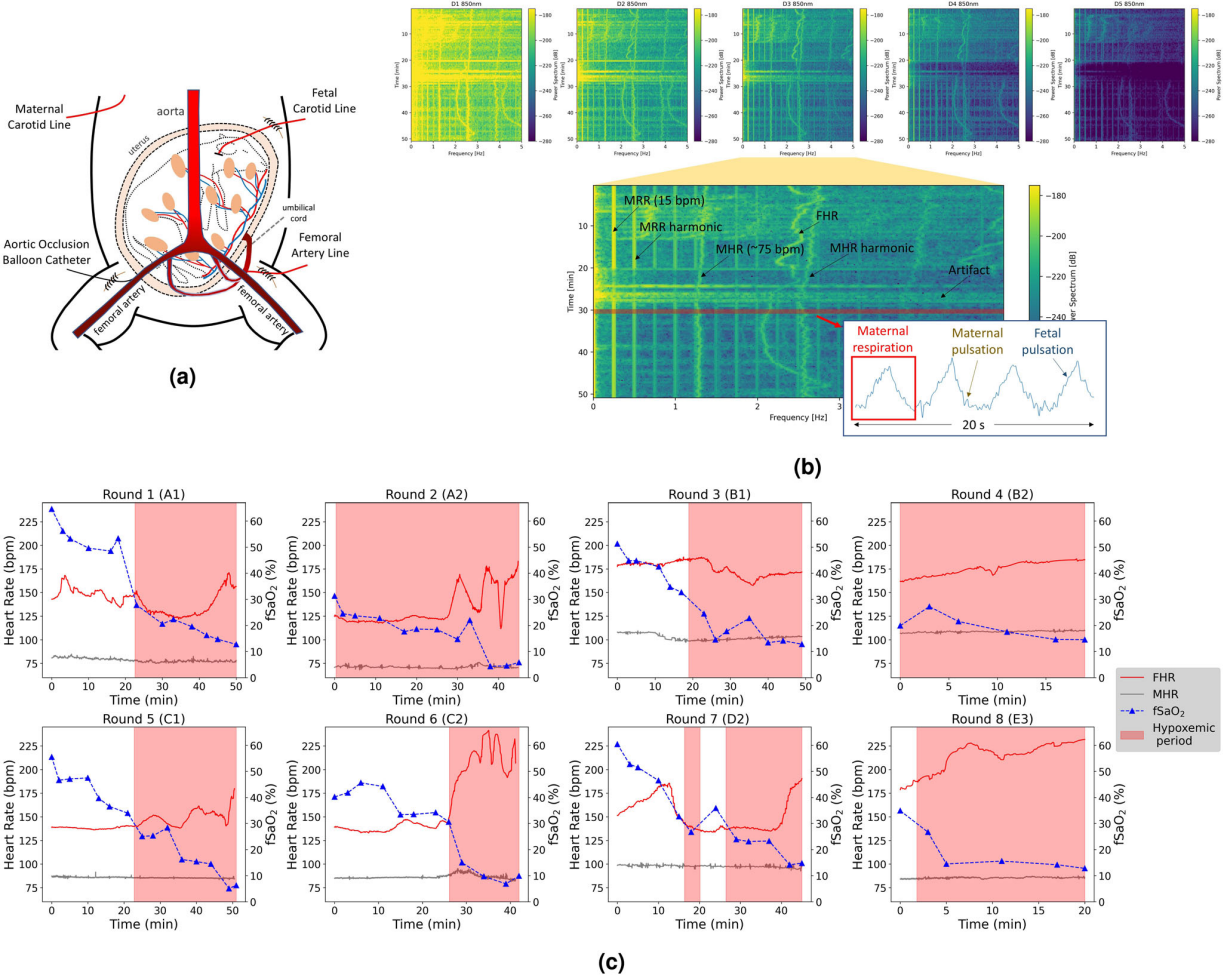
Experiment of pregnant ewe with hypoxic in-utero fetal lamb model used in this study. **a** Sketch of pregnant ewe with hypoxic in-utero fetal lamb model. The study protocol is discussed in section “Methods”. **b** Short-time Fourier transform (STFT) of the mixed 850nm PPG collected by five detectors. The spectrogram of detector 3 is zoomed-in, and labeled with major signal components. A 20-s time series is also shown. **c** fSaO_2_ and heart rate readings from *n* = 8 hypoxic rounds are used in this study. Each subplot denotes fSaO_2_ readings along with maternal and fetal heart rates for the full hypoxic round starting from the maternal dMAP is regulated to induce fetal hypoxia. Triangles denote the timestamp of collected fetal blood samples and the corresponding value of fSaO_2_. The blood samples were nominally drawn at 2.5, 5, and 10 min mark within each 10-min hypoxic step. The red background denotes hypoxemic periods, defined as fSaO_2_ being below 30% after linear interpolation of fetal blood ABG data. The letter in a subplot title indicates the individual sheep, while the number represents the specific hypoxic round within each experiment. For example, “Round 3 (B1)” refers to the first round of experiments conducted on sheep B.

We conducted experiments on five pregnant ewes with a hypoxic in-utero fetal lamb animal model of pregnancy (Fig. 2a), which is elaborated in the section “Methods”. Five pregnant sheep were used at 137 ± 3.9 days of gestational age. Each experiment contained one or more hypoxic rounds. In each round of an experiment, fetal hypoxemia was induced through resuscitative endovascular balloon occlusion of the Aorta (REBOA) above the ewes’ iliac bifurcation. Arterial Blood Gas (ABG) analysis was performed on fetal blood samples to obtain the ground truth fetal arterial blood oxygen saturation (fSaO_2_). Reference maternal and fetal heart rate data were collected through hemodynamic monitoring. After excluding rounds with a low fetal signal-to-noise ratio in which, either the FHR was not discernible in the PPG signal spectrum, or they contained excessive signal artifacts masking the FHR, a total of *n* = 8 hypoxic rounds from five animal surgeries were selected for this study (Fig. 2c). Three sheep contributed two rounds (sheep A, B, and C) and two sheep contributed one round (sheep D and E) in this study after selection. FSaO_2_ decreased during each round due to the gradual inflation of the balloon catheter and decreased distal blood pressure. The initial value of fSaO_2_, and the temporal length of each hypoxic round varied due to inter-subject physiological variation. Five rounds started with an initial fSaO_2_ value between 35 and 60%, and three rounds started between 15 and 30%. The initial hypoxic rounds in one surgery typically lasted about 40–50 min, while subsequent rounds of the same experiment were notably shorter. The observed FHR in some rounds suggests that the fetal peripheral chemoreflex may have been affected by the anesthetic protocol used^22,23^. However, the collected blood samples remain sufficient to serve as ground truth for this study.

The TFO device was placed on the ewe’s abdomen during each hypoxic round to collect mixed-PPG signals. The collected PPG signal comprises a mixture of maternal and fetal signal components, along with noise and artifacts arising from various sources, including occasional fetal movements, manipulation of the animal by the surgical team, and electronic measurement noises. The primary signal components in the acquired mixed signal are due to maternal respiratory rate (MRR), maternal heart rate (MHR), and fetal heart rate (FHR). Figure 2b illustrates spectrograms of the mixed-PPG signals collected at 850nm by photodetectors placed at varying distances from the light source in a 50-min hypoxic round. In this example, MRR exhibits a constant frequency at 0.25 Hz (15 bpm) due to the fixed respiratory rate administered by the ventilator, which creates strong harmonics in the PPG spectrograms. During this hypoxic round, MHR decreases from 1.4 Hz (84 bpm) to 1.25 Hz (75 bpm), while FHR varies over a larger range between 2 Hz (120 bpm) and 2.9 Hz (174 bpm). Figure 2c shows *n* = 8 hypoxic rounds used in this study. The average time length is 34.5 min. As shown in Fig. 2b, detectors closer to the light source receive higher total optical power compared to farther detectors, however the share of fetal signal in the sensed signal by closer detectors is far smaller than that of farther detectors. It is noteworthy that while fetal signal has a better signal-to-noise ratio (SNR) in detectors 2, 3, and 4 in this specific round, in general, the signal correlation among detectors varies, since the fetal depth and measurement geometry differ significantly across subjects. This implies that the quality of PPG signal acquired by each detector and the relative importance of each detector for fetal assessment vary across subjects, and may also vary dynamically over time for the same subject.

### Extraction of fetal pulsation ratio and modulation ratio from mixed PPG

**Fig. 3:**
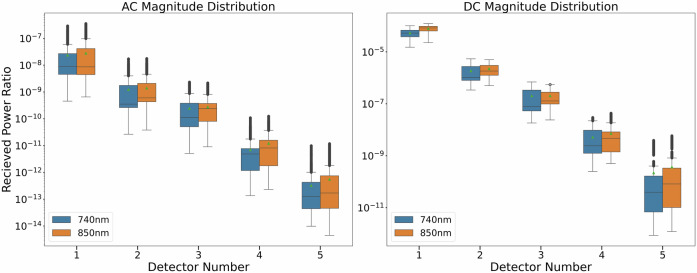
Distribution of AC and DC magnitude of the PPG data sensed by each detector at the two wavelengths. The box plot shows the inter-quartile range of values for each detector channel with two whiskers. Outliers are shown as black dots outside whiskers. The green triangles and mid-line denote the mean and median of the distribution, respectively. The y-axis shows the range of the received power ratio in the log scale.

We extracted the alternating current (AC) and direct current (DC) components of the source-normalized detected optical power through lock-in detection and signal enveloping, respectively. Figure 3 presents the distribution of AC and DC for five detectors at two wavelengths across all rounds. The distribution of the source-normalized received optical power ratio varies across detectors, exhibiting an exponentially decreasing trend as the light source-detector distance increases, consistent with the Beer–Lambert law. This indicates that the fraction of emitted photons hitting the photodetector exponentially decreases as more photons are scattered away or absorbed by tissue over longer distances. The AC magnitude is consistently smaller than the DC magnitude by two to four orders of magnitude, reflecting the relatively small change in tissue absorption due to fetal pulsation, compared to non-pulsatile maternal and fetal tissue layers.

**Fig. 4:**
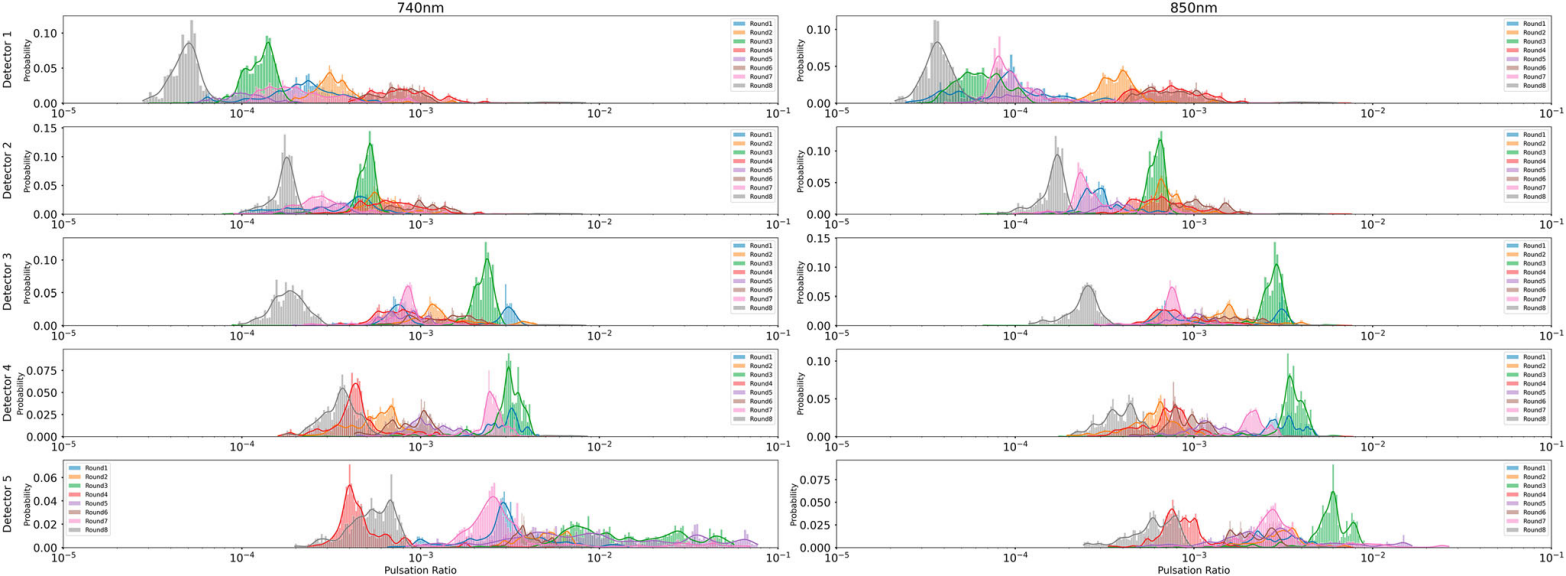
Probability distribution of AC/DC ratio data. Subplots display the probability distribution of normalized pulsation for subjects. Rows indicate detectors 1 to 5, and two columns correspond to the two measurement wavelengths.

The wavelength-specific pulsation ratio is defined as AC divided by DC, and its distribution is displayed in Fig. 4. A more concentrated distribution of pulsation ratios implies better stability, while a wider distribution indicates inter-subject variability, potential DC drift, or AC contamination due to disturbances, such as fetal motion. In the same round, the pulsation ratio increases from detectors closer to the light source to farther detectors since only a small fraction of photons collected by near detectors travel through the fetal layer.

Subsequently, the modulation ratio is calculated by computing the ratio of pulsation ratio at 740 nm to the pulsation ratio at 850 nm following smoothing and outlier rejection filters. Specifically, modulation ratios out of range [0.01, 100] were rejected as outliers. From *n* = 8 hypoxic rounds shown in Fig. 2c, the total number of samples used in the following step is 16,563. Each sample contains ten pulsation ratios and five modulation ratios values, associated with the two wavelengths and five detectors. The range of pulsation ratio captured by the same detector differs for each round due to anatomical, physiological and sensor placement variations, thus the combination of 10 pulsation ratio is included in each sample as a signature of the measurement context.

### Detection of fetal hypoxemia through multi-detector signal fusion

A multi-layer perceptron (MLP) network was developed for the detection of fetal hypoxemia from dual-wavelength multi-detector transabdominal PPG signals. MLP performs binary classification of its input samples, defining hypoxemia cases as positive and normoxemia as negative. The hypoxemia threshold was adopted at 30%, as suggested by clinical benchmarks and existing literature^24–28^. A custom fivefold cross-validation method was developed to assess model performance, and the details of which are discussed in section “Methods”.

**Fig. 5:**
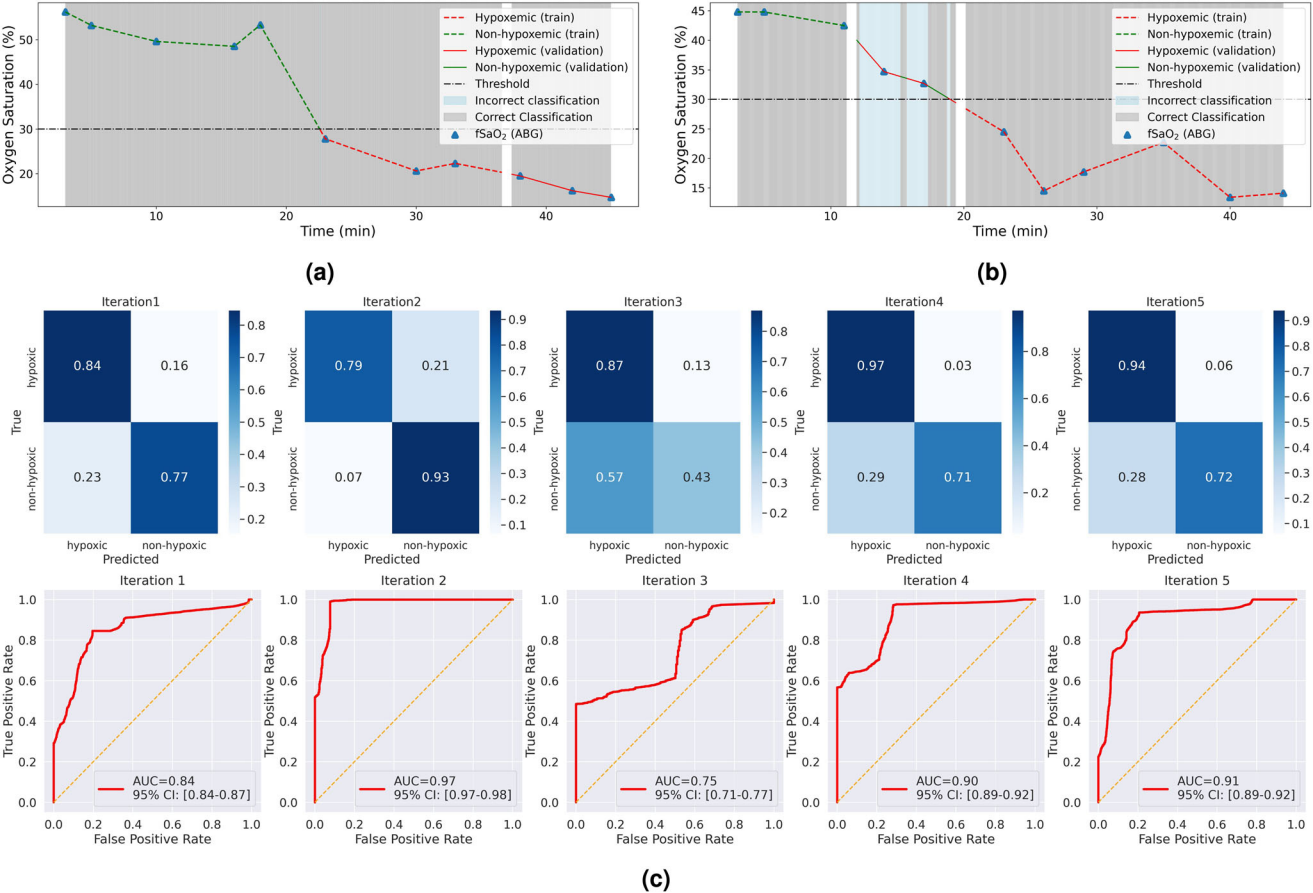
The binary classification demonstration and results of fivefold cross-validation. **a**, **b** Demonstration of continuous classification from two hypoxic rounds. In one iteration of cross-validation, 80% of the data from each round contributes to the training set, while the remaining 20% is used for validation. After excluding low-quality samples, the validation samples for **a** are from 37 to 42 min, and for **b** are from 12 to 19.5 min. **c** Confusion matrix and ROC curves for each shuffle in fivefold cross-validation. Each shuffle uses fourfold continuous samples for training, and onefold for validation. The ROC curves treat hypoxemia as positive results and normoxemia as negative results.

Given the variability in duration and fetal oxygen saturation range in different experiments, the numerical frequency of positive and negative samples varies across each iteration, as detailed in Table 1. The model’s performance is evaluated using a suite of metrics, including accuracy, sensitivity, specificity, precision, and the F1 score. Accuracy represents the proportion of correctly classified validation samples, yielding results across cross-validation shuffles as follows: [84, 87, 86, 93, and 88%], averaging an 87.6% accuracy rate. Figure 5a, b show the continuous classification results for round 3 and round 7 in the first iteration. Both rounds lasted 42 min. The gray background indicates regions of correct classification, while the light blue denotes incorrect classification. In this instance, round 3 achieved 100% accuracy, while round 7 achieved 48% accuracy, both based on validation samples only.

**Table 1:** Performance of detecting fetal hypoxemia in five cross-validation shuffles

Iteration	Hypoxemic samples (validation)	Hypoxemic samples (training)	Normoxemic samples (validation)	Normoxemic samples (training)	Accuracy	Sensitivity	Specificity	Precision	*F*1 score
1	1238	8246	1234	2413	0.84	0.84	0.77	0.79	0.82
2	1943	7406	484	3253	0.87	0.79	0.93	0.98	0.87
3	2200	7103	227	3600	0.86	0.87	0.43	0.94	0.90
4	1992	7391	480	3313	0.93	0.97	0.71	0.93	0.95
5	1374	8143	1098	2560	0.88	0.94	0.72	0.81	0.87

The confusion matrices and receiver operating characteristic (ROC) curves are summarized in Fig. 5c. The ROC curve analysis corroborates the model’s discriminative prowess, with AUCs recorded at [0.84 (CI:0.84–0.87), 0.97 (CI:0.97–0.98), 0.75 (CI:0.71–0.77), 0.90 (CI:0.89–0.92), 0.91 (CI:0.89–0.92)], and average AUC at 0.87.

To address the potential bias introduced by an imbalanced validation set, sensitivity (true positive rate) and specificity (true negative rate) were computed. The classifier demonstrates strong sensitivity across all iterations, correctly identifying hypoxemia samples at rates of [84, 79, 87, 97, and 94%]. The specificity exhibited more variability, with true negative rates of [77, 93, 43, 71, and 72%] for normoxemia sample classification. Across the shuffles, the true positive rates remained consistently high, averaging 88.2%. These results affirm the model’s overall effectiveness, particularly in detecting hypoxemia conditions, thereby demonstrating its potential to supplement the standard of care for intrapartum fetal monitoring.

## Discussion

This study demonstrates the feasibility of non-invasive, continuous detection of fetal hypoxemia in gold standard large animal model of pregnancy. The approach of information fusion across multiple detectors represents a significant advancement over prior art^19,21^, and enables both continuous and robust monitoring across a number of subjects, which were not possible in the past. Incorporation of information sensed across all detectors accomplishes two goals: first, it reduces dependence on the signal quality of a single detector. More importantly, it enables resolving the ambiguity between fetal depth and fetal oxygen saturation, both of which influence the detected optical signal at a detector. As a result, the two variables cannot be deterministically isolated using a single detector PPG signal.

Mixed-PPG signals are processed to derive both fetal AC and DC components. Subsequently, pulsation and modulation ratios are computed, and fed into a fSpO_2_ inference model, which learns the appropriate weight for each detected signal from the data, without a need for prior knowledge of the relative importance of each detector. By adopting round-based cross-validation, we have demonstrated the algorithm’s robust performance, achieving an average accuracy of 87.6%, sensitivity of 88.2%, specificity of 71.2%, precision of 89%, and an F1 score of 88.2%. This demonstrates strong classification capabilities for non-invasive detection of fetal hypoxemia in-utero, and highlights the promise of the approach to supplement the standard of care for intrapartum fetal monitoring.

The main challenge in the non-invasive detection of fetal hypoxemia arises from signal quality, which significantly impacts classification accuracy. Our method leverages lock-in detection to isolate variations in the received mixed-PPG signal that are due to fetal arterial pulsation (fetal AC). This approach is susceptible to contamination from maternal signal components or other noise sources, which may have a spectral component that overlaps with the fetal heart rate. Similarly, non-fetal-related artifacts may render the signal DC band unstable. Furthermore, the potential movements of the fetus during a measurement can dynamically alter the signal-to-noise ratio (SNR) of fetal signal.

**Fig. 6:**
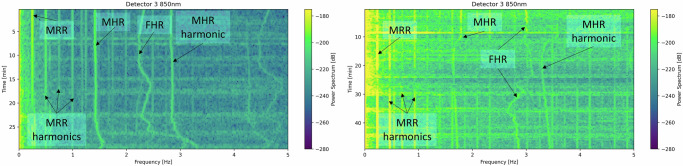
Comparison of PPG signal time-frequency (spectrogram) plots in two different hypoxic rounds. The two spectrograms are generated from the same detector at 850 nm. The left experiment exhibits better SNR, while there are more noise, motion artifacts, and environmental disturbances present in the right experiment. The fetal pulsation is consistently observable in the left plot, however, it fades away at 18-min into the right experiment, and becomes discernible again at 30-min.

The quality of mixed-PPG signals varies across hypoxic rounds, resulting in samples with differing information content. For example, the right spectrogram in Fig. 6 shows strong motion artifacts and disturbances to the measurement due to the blood draws. These artifacts, including touch and pressure in the soft tissue around the measurement area, are unpredictable and often unavoidable, leading to large drifts in detected signal amplitude. The sudden change in the temporal profile of the signal manifests itself as large spectral content in many frequency bins of the PPG signal spectrum. We have made efforts to maintain sample quality, e.g., via implementing outlier rejection and signal truncation before calculating modulation ratios. However, the extent of contamination and information loss in the processed data samples, both in the AC and DC components of the signal, remain challenging to quantify.

Another challenge is the size of the dataset available to train the fSpO_2_ inference model, which limited the scope of machine learning models that we could utilize. Variability in duration of hypoxic rounds (from 21 to 51 min), and difference in fSaO_2_ ranges lead to an imbalanced dataset of hypoxemia and normoxemia samples in each training iteration. Although techniques such as the assignment of higher weights to the minority class during training are utilized, it must be acknowledged that the model had access to fewer distinct non-hypoxic samples during training.

As shown in Fig. 2c, four rounds contain both non-hypoxemic (>30%) and hypoxemic samples, and four rounds contain only hypoxemic samples. We have partitioned data from each round for each iteration to ensure that both training and validation sets contain some number of non-hypoxemic samples. Ideally, a more expansive dataset, encompassing diverse sensing scenarios, a larger cohort of subjects, and a collection of wider ranges of fSaO_2_, would be available to train the fSpO_2_ inference model. Such a dataset would enable the network to learn a more representative understanding of the complex dynamics involved in transabdominal fetal oximetry, and to extrapolate learned features from the training set to unseen scenarios. Furthermore, this study serves as a proof-of-concept for continuous fSpO_2_ measurement and is defined as a binary classification task with an oxygen saturation threshold of 30%. We recognize that the classification results do not reflect the complexity of fetal asphyxia that occurs in labor, and the current dataset lacks sufficient data to train a model capable of inferring the true fSpO_2_ levels, which would have greater clinical significance. Achieving accurate fSpO_2_ readings is a future goal for the TFO system.

The study protocol was designed with the aim of supporting TFO system validation for the detection of instantaneous hypoxemia. We recognize that the adopted protocol may not reflect the majority of HIE cases, which typically involve evolving hypoxia^29^. Establishing the potential value of fetal instantaneous hypoxemia, the parameter sensed by the TFO system, as a biomarker for the detection of fetuses at risk of HIE requires further investigations.

The existing standard of practice based on CTG trace interpretation guidelines are subject to significant inter-observer and intra-observer variability^11,30–32^. Another noteworthy study has underscored the lack of consensus among the guidelines for the diagnosis of fetal distress^33^. TFO, as a non-invasive and continuous method, has the potential for the detection of fetal hypoxemia, which fundamentally addresses the issue of reliance on fetal heart rate as a surrogate measure of fetal hypoxic distress. Our proposed system provides a high level of sensitivity, while offering a decent specificity (>70%) in four out of five cross-validation shuffles and high precision (>75%) across all shuffles. Given that the performance of machine learning-based fSpO_2_ inference model is expected to improve with availability of additional data, transabdominal fetal oximetry presents a viable tool for complementing standard of care for detection of intrapratum fetal hypoxemia.

## Methods

### Background on conventional pulse oximetry

The cardiac cycle induces a periodic small change in blood-to-tissue ratio at the extremities, manifesting as an increase during systole and a decrease during diastole cycles, respectively. Oxyhemoglobin (O_2_Hb) and deoxyhemoglobin, also referred to as reduced hemoglobin (RHb), have different optical absorption properties in red and near-infrared regions of the electromagnetic spectrum. Consequently, the dynamics of the blood-to-tissue ratio cause temporal changes in the red and near-infrared light absorption properties of the tissue. For a given measurement geometry, the amount of light absorbed by the tissue decreases in the diastolic phase compared to the systolic phase. Therefore, the detected light has higher intensity during the diastolic phase compared to the systolic phase.

**Fig. 7:**
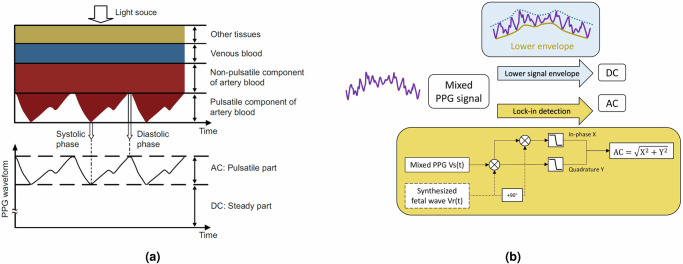
AC/DC definition in PPG signal and the method used to extract AC/DC in this study. **a** A demonstration of how PPG signal acquires information of arterial pulsation. Arterial pulsation causes temporal changes to the light absorption, which is sensed as a PPG signal. Figure courtesy of Sun et al.^39^. **b** The signal lower envelope forms the DC of mixed PPG. Lock-in detection enables the extraction of the PPG signal component at the FHR frequency. Specifically, the input signal *V*_*s*_(*t*) undergoes mixing with both a reference sine wave at the FHR frequency, and its 90° phase-shifted wave separately. Subsequently, the mixer outputs are low-pass filtered to attenuate signal components at frequencies other than the desired FHR frequency. The amplitude *V*_*o*_(*t*) is derived by transforming the information from Cartesian coordinates into polar coordinates.

Conventional pulse oximetry takes advantage of this phenomenon to infer the subject’s arterial blood oxygen saturation from non-invasively acquired light intensity measurements. The acquired PPG signals consist of two components: the varying AC component arising from the temporal fluctuation of blood volume in the light path, and the stable DC component resulting from the temporally-static chromophores, such as non-pulsatile part of the arterial blood, as well as non-pulsatile tissues, such as veins, capillaries and other tissues with negligible blood volume change (Fig. 7a). The change of light absorbance for a given wavelength (*λ*) between two close times *t*_0_ and *t*_1_ then can be described with modified Beer–Lambert law (mBLL):

1 $$\begin{array}{ll}\Delta {A}^{\lambda }=-{\log }_{10}\frac{{I}_{{t}_{1}}^{\lambda }}{{I}_{{t}_{0}}^{\lambda }}=\sum _{i}\Delta {C}_{i}\cdot {\varepsilon }_{i}\cdot \langle L\rangle\\\qquad\qquad =\left(\Delta {C}_{{O}_{2}Hb}\cdot {\varepsilon }_{{O}_{2}Hb}^{\lambda }+\Delta {C}_{RHb}\cdot {\varepsilon }_{RHb}^{\lambda }\right)\cdot \langle L\rangle\end{array}$$

Δ*C* denotes the change in concentration of the absorbing molecules (in mM); *ε* is the extinction coefficient (in molar^−1^ cm^−1^); index *i* denotes the number of chromophores (here assuming O_2_Hb and RHb are two major chromophores). 〈*L*〉 is the mean photon path length.

The oxygen saturation (S) is commonly expressed as the percentage of hemoglobin molecules (THb) that are bound to oxygen (O_2_Hb). It follows that $S={C}_{{O}_{2}Hb}/{C}_{THb}={C}_{{O}_{2}Hb}/({C}_{{O}_{2}Hb}+{C}_{RHb})$. Thus, by taking the ratio (Φ) of the change in optical absorbance at two wavelengths, we can derive the S from Φ with other constant coefficients:

2 $$\begin{array}{l}\Phi =\frac{\Delta {A}^{{\lambda }_{1}}}{\Delta {A}^{{\lambda }_{2}}}=\frac{\left[{\varepsilon }_{{O}_{2}Hb}^{{\lambda }_{1}}\cdot S+{\varepsilon }_{RHb}^{{\lambda }_{1}}\cdot (1-S)\right]\cdot \Delta {C}_{THb}\cdot {\langle L\rangle }^{{\lambda }_{1}}}{\left[{\varepsilon }_{{O}_{2}Hb}^{{\lambda }_{2}}\cdot S+{\varepsilon }_{RHb}^{{\lambda }_{2}}\cdot (1-S)\right]\cdot \Delta {C}_{THb}\cdot {\langle L\rangle }^{{\lambda }_{2}}}\,\mathop{\to }\limits^{{\langle L\rangle }^{{\lambda }_{1}}\approx {\langle L\rangle }^{{\lambda }_{2}}}\\S=\frac{\Phi \cdot {\varepsilon }_{RHb}^{{\lambda }_{2}}-{\varepsilon }_{RHb}^{{\lambda }_{1}}}{{\varepsilon }_{{O}_{2}Hb}^{{\lambda }_{1}}-{\varepsilon }_{RHb}^{{\lambda }_{1}}-\Phi \cdot \left({\varepsilon }_{{O}_{2}Hb}^{{\lambda }_{2}}-{\varepsilon }_{RHb}^{{\lambda }_{2}}\right)}\end{array}$$

This derivation assumes single body setup in which, the pulsating arteries are primarily responsible for the change in detected light intensity. In addition, the tissue between the light source and detector is assumed to be relatively thin, and as a result, both wavelengths are expected to have similar path lengths in the tissue.

In practice, the temporal changes in tissue absorbance are far smaller than its static absorbance. Thus, the ratio Φ can be approximated with two amplitudinal ratios at two wavelengths:

3 $$\Phi \approx (A{C}^{{\lambda }_{1}}/D{C}^{{\lambda }_{1}})/(A{C}^{{\lambda }_{2}}/D{C}^{{\lambda }_{2}})$$

### Transabdominal fetal oximetry

Transabdominal fetal oximetry (TFO) refers to the concept and technology for non-invasive measurement of fetal arterial blood oxygen saturation through the maternal abdomen of a pregnant person. TFO leverages the same underlying physical property that enables conventional pulse oximetry, i.e., the difference in absorption spectra of fetal hemoglobin in oxy- and deoxy- states. Although fetal and adult hemoglobin are different molecules with different affinity for oxygen, their optical properties in the red and near-infrared regions of the spectrum are quite similar^34,35^. Typical fetal oxygen saturation levels are in the 30 to 70% range^24,36^, and as a result, conventional wavelength choices for conventional pulse oximetry, such as 660 and 940 nm, yield inferior results for TFO^37^.

For TFO to function non-invasively, some light photons need to travel deep enough into maternal body to interrogate fetal tissue, and then diffuse back to the outside of the maternal abdomen to be sensed. Such subset of photons are impacted by temporal changes of both fetal and maternal blood-to-tissue ratio, and thus, the associated sensed signal would carry a mixture of maternal and fetal information. Another subset of photons may reach the detectors without going through the fetal tissue. We use the term mixed signal and maternal signal to refer to the signal generated by the former and latter group of photons, respectively. Inference of fetal blood oxygen saturation from such a mixture of acquired signals is a foundational technical challenge in TFO, which is exacerbated by the exponential attenuation of light with traveled path length in tissue.

Our team has designed a novel TFO system prototype featuring two light sources emitting non-coherent light at 740nm and 850nm wavelengths, modulated at 690 and 940 Hz, respectively. The sensor also features five detectors positioned at distances of {1.5, 3, 4.5, 7, 10}cm from the LED light sources^19,20^. Detectors at varying distances acquire signals from photons with a different distribution of pathlength, with the expectation that farther detectors receive exponentially weaker signals from photons that have, on average, penetrated deeper into the body. The TFO sensor also features adjustable gain values for detectors, and driving currents for LED light sources, allowing for precise subject-specific control of radiated light energy into the body. In previous studies^21,38^ (Fig. 1), we utilized a single-detector PPG signal and subject-specific calibration to provide intermittent fSpO_2_ measurements. In this paper, we present a novel data processing pipeline, which fuses information collected across detectors to offer a subject-agnostic continuous detection of fetal hypoxemia.

### Pregnant ewe with in-utero hypoxic fetal lamb model

A pregnant ewe with an in-utero hypoxic fetal lamb model was used to demonstrate the promise of TFO for continuous fetal monitoring. The study protocol was approved by the Institutional Animal Care and Use Committee (IACUC), numbered 22476, at the University of California, Davis. We conducted controlled fetal hypoxemia surgeries on five time-mated pregnant ewes, with a gestational age of 137 ± 3.9 days.

In each surgery, the pregnant ewe was placed under general anesthesia, maintaining a steady respiration rate through mechanical ventilation using a 100% fraction of inspired oxygen (FiO_2_). An endovascular balloon catheter was inserted through the femoral artery and advanced to the abdominal aorta past the iliac bifurcation. The location of the balloon was confirmed through fluoroscopy. The objective of this procedure was to enable controlled induction of fetal hypoxemia, via controlled regulation of the ewe’s aortic distal blood pressure.

Then hysterotomy was performed, and the fetal lamb’s head was exteriorized. A fetal carotid arterial line was placed for monitoring of fetal hemodynamics and fetal blood sample draws. Subsequently, the fetus was returned to the uterus, and the lost amniotic fluid was replaced with warm saline. To secure the position of the fetus in-utero, one of the lamb’s ears was sutured to the inside of the uterus to minimize its movement. The ewe’s uterus and fascia were then closed. The position of the fetus in-utero was relatively stable, while sometimes external pressure was applied to facilitate blood draws which may have slightly changed the fetal position. After the surgical steps were completed, the TFO probe was placed on the ewe’s closed abdomen, such that the device light emitters were directly above the fetal head. Prior to each experiment, the gain of optical detectors and LED driving current were empirically selected, and adjustments were made during each experiment as necessary. A visual representation of the animal model is depicted in Fig. 2a.

For each experiment, fetal hypoxemia was systematically induced by incrementally inflating the aortic balloon catheter, causing a stepwise reduction in maternal distal Mean Arterial Pressure (dMAP). This gradual reduction in dMAP resulted in a corresponding decrease in the amount of oxygen transported from the ewe to the fetus. The first hypoxic step targeted a dMAP of 50 mmHg. If the baseline dMAP was below 50 mmHg, the closest multiple of 5 mmHg below the baseline dMAP was targeted. In each subsequent step, the dMAP was reduced by 5 mmHg until the termination criteria was reached. Each hypoxic step was sustained for ten minutes, during which three blood samples were extracted at 2.5, 5, and 10 min for subsequent ABG analysis (Radiometer ABL 90 Flex). These ABG recordings were used as the ground truth of the fSaO_2_. Each hypoxic round concluded when the maternal dMAP reached 20 mmHg, or if two consecutive fetal ABG readings were below 15%. In practice, all experiments ended due to the latter condition. In the experiment (Fig. 2c), some rounds had more than two ABG readings below 15%, since the ABG analysis on the fetal blood sample might have been delayed but the experiment was still continuing concurrently. A 45-min recovery interval was scheduled after a hypoxic round during which, the balloon catheter was fully deflated and the fetus was given time to recuperate. ABG analyses were conducted at 30 and 45 min into the recovery phase. If, following the recovery time, fSaO_2_ remained below 15%, the subsequent hypoxic round would not be initiated. In cases where the fetus recovered to fSaO_2_ higher than or equal to 15%, a maximum of three hypoxic rounds were conducted. Following the completion of the experiment, the fetal lamb and ewe were euthanized in adherence to the approved protocol guidelines. More information on the sheep experiment can be found in supplementary material.

### Mixed photoplethysmogram signal processing

Silicon photodetectors are color-blind, and their output is influenced by a fairly large range of input light wavelengths. In the TFO system, as a result, each detector output, sampled at a rate of 8k samples per second (sps), carries an aggregate of signals associated with two different light wavelengths. To separate the signals associated with each wavelength of light, the two light sources are toggled on and off at different 690 and 940 Hz rates. Subsequently, a demodulation process is implemented to extract wavelength-specific mixed-PPG signals from each detector output. The resulting signal is further downsampled from 8 kHz to 80 Hz to improve its SNR, and to enhance computational efficiency.

Since in each experiment, different device configuration parameters, such as light source intensity or photodetector output gain, could have been used, the acquired signals are converted to source-normalized detected optical power. The conversion ensures that signals collected from different subjects or rounds are directly comparable, effectively enabling cross-subject model calibration and analysis.

In conventional single-body pulse oximetry, the classic algorithm for oxygen saturation inference involves calculating the pulsation ratios at two distinct wavelengths, and calibrating the ratio of pulsation ratios, referred to as the modulation ratio (also known as the ratio of ratios), against reference arterial blood oxygen saturation (SaO_2_) values. We adopt a similar approach by extracting both the AC and DC components of the PPG signal, and subsequently calculating the pulsation ratio as well as the modulation ratio at each detector.

The DC component of the mixed-PPG signal originates from a composite of static maternal and fetal tissues, such as a non-pulsating component of arterial blood, veins, and capillaries of both the mother and baby, adipose tissue, and amniotic fluid. The DC component is obtained via characterization of the lower envelope of the mixed-PPG signal, which is implemented via identification of local extrema in timer series, followed by interpolation of such points. The AC component was extracted using a lock-in detection algorithm, which selectively isolates the signal at the frequency of interest from a noisy background, effectively rejecting all other frequency components. In our application, the frequency of interest is the FHR, which was measured through hemodynamic monitoring of the fetal carotid arterial line in the experiment. The temporal profile of FHR was utilized to generate a variable-frequency sine wave as the reference signal in the lock-in detection algorithm. The process of extracting the AC and DC components, as well as the lock-in detection algorithm, are visually depicted in Fig. 7b.

Subsequently, the wavelength-specific pulsation ratio for each mixed-PPG is obtained by dividing the AC component by the DC component of the signal. The modulation ratio is computed by dividing the pulsation ratio of 740 nm wavelength by that of 850 nm wavelength using the equation (3). Samples whose modulation ratio (Φ) was outside the specified range of [0.01, 100] were deemed to be outliers and discarded. The decision is based on the assumption that pulsation ratios at the two wavelengths should be at most 2 orders of magnitude apart, or equivalently, that larger differences indicate strong disturbances to the measurement that that have corrupted the signal. Following outlier rejection, modulation ratios were filtered via averaging over a 1.5-min window of non-outlier data points. The filtering process reduces the contribution of high-frequency noise, and enhances the robustness and stability of modulation ratio estimation.

### Classification via information fusion across detectors

In addition to the fetal arterial blood oxygen saturation, the signals measured at each detector are influenced by a number of other anatomical and physiological parameters, such as fetal depth and maternal tissue composition. As a result, resolving fSpO_2_ from the measurements taken at a single detector faces ambiguity. We propose to solve this problem by integrating information that is concurrently collected across a number of detectors, and use the term information fusion to refer to the concept. Specifically, we have developed a deep neural network architecture that fuses information collected across detectors to classify input samples into the two hypoxemia or normoxemia groups.

**Fig. 8:**
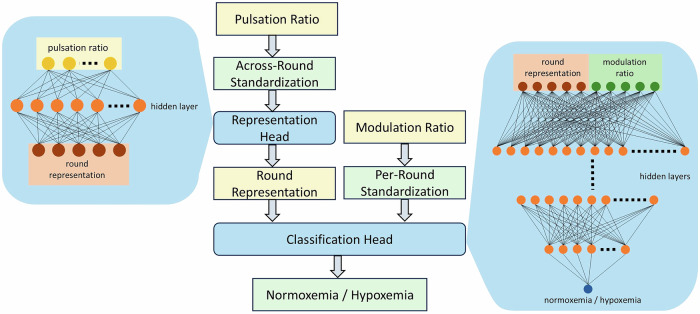
Overview of the MLP network for information fusion across detectors. The model contains a representation head, which takes as input normalized pulsation ratio measured across all detectors, and generates the proper data representation for a certain round. The classification head interprets the normalized modulation ratio across detectors in the context of round representation to determine the detection outcome.

The network architecture consisted of two components: a representation head and a classification head (see Fig. 8). The representation head features one linear layer followed by a nonlinear rectified linear unit (ReLU) activation, while the classification head contains multiple linear layers, each followed by a ReLU activation function. The number of neurons in each subsequent layer was halved until it reached 16 output neurons. Upon tuning the network architecture, the representation head featured one layer with 16 neurons, while the classification head comprised four hidden layers starting from 128 neurons.

Two sets of input data are provided to the model: ten pulsation ratio readings from five detectors at two wavelengths, and five outlier-rejected modulation ratios computed independently for each of the five detectors. The pulsation ratio represents each round in feature space, while the modulation ratio captures the impact of fSpO_2_ on the signals measured at the two wavelengths. Serving different purposes in the network, different normalization schemes were applied to pulsation ratios and modulation ratios. Pulsation ratios, encapsulating information on light attenuation for a particular subject and measurement geometry, were standardized across rounds. That is, pulsation ratios measured at a specific detector and wavelength, are scaled to have zero mean and unit variance across all rounds. On the other hand, outlier-rejected modulation ratios, measured at each detector, are standardized for each round separately, as they exhibit wide subject-specific sensitivity to fSpO_2_, as a function of the unique anatomical features, probe placement and consequent measurement geometric adopted in each experiment.

### Model training and validation

The post-processing step generates modulation ratios at the rate of 1 sps. Each modulation ratio value is estimated from a window of raw data around that timestamp. Thus, random assignment of modulation ratio data into training and validation sets would risk leaking information from the training to the validation set, and would introduce bias. Stated differently, similar temporal information is potentially contained in adjacent samples. To address this issue, we adopt a round-based cross-validation scheme in which, each round data is partitioned into five folds with respect to the time. In each iteration, one fold from each round’s data serves as validation samples, and the remaining four folds are used as training samples. Training samples whose averaging window may overlap with those of the validation samples are discarded to avoid the introduction of bias.

#### Algorithm 1

Formation of training and validation sets

* p* = [0%, 20%. . . , 80%, 0%, 20%. . . ] ⊳ Start percentage for validation for each round.

** for** *j* in iteration (0, 4) **do**

* n* = (*p* + 20%$*$j) *mod* 100% ⊳ Start percentage shift for each iteration.

** for** each round *r* **do**

 ${X}_{r}=\mathop{\sum }\nolimits_{i = 0}^{l}{x}_{i};$ ⊳ Round *r* contains *l* samples.

* X*_*V*_ = {*x*_*i*_, *n*[*r*]$*$l < *i*≤(*n*[*s*] + 0.2)*l}; ⊳ 20% of samples are taken as validation samples.

 ${X}_{T}={X}_{V}^{C};$ ⊳ Complement of the validation set, excluding the overlapping samples, are taken as training samples.

* U*_*V*_ ← *X*_*V*_, (*X*_*V*_ ⊆ *X*_*r*_); ⊳ Validation samples for round *r* are added into the validation set.

* U*_*T*_ ← *X*_*T*_, (*X*_*T*_ ⊆ *X*_*r*_); ⊳ Training samples for round *r* are added into the training set.

**end for**

 {*U*_*T*_, *U*_*V*_} for training and validation

**end for**

Our animal experiment design involves progressive inflation of the endovascular balloon as a result of which, all subjects exhibited a general trend of decreasing fSaO_2_ during a hypoxic round. It follows in forming training and validation sets, one needs to ensure that both training and validation samples contain a representative set of examples at different fSaO_2_ values.

To enhance model generalizability during each training iteration, we select folds for the training set to ensure that a large enough range of oxygen saturation labels are revealed to the model during training, while the fold included in the validation set varied for each round. For example, the validation set of the first iteration included the 1st fold in round 1, the 2nd fold in round 2, and so on. This strategy ensured that samples with a larger range of oxygen saturation labels are included in both training and validation in each iteration. Furthermore, the next iteration used the (*n* + 1)th fold for validation if the current iteration used the *n*th fold in validation for each round. This approach mitigated issues such as selection bias and provided insights into how the model was generalized in this application. The selection of training and validation sets is summarized in Algorithm 1.

Training of the neural network aimed to minimize the Binary Cross Entropy loss function. Different weights are assigned to two hypoxemia and normoxemia classes to counter the imbalance between the number of positive and negative samples in the training set. The Adam optimizer with an initial learning rate of 1e-4, and weight decay coefficient of 1e-4 is employed to prevent overfitting. Initial weights of the neural network were randomized with a normal distribution, and bias values were initially set to zero. The training process consisted of 300 epochs, with an early stoppage strategy implemented to halt training if validation error did not improve for 25 epochs, restoring the best model weights for inference. This network and training process were implemented using PyTorch 2.1.

## Supplementary information

Supplementary Information

## Data Availability

The data used in this study were obtained at University of California, Davis. Data were available upon request to wtqian@ucdavis.edu or ghiasi@ucdavis.edu.

## Code availability

The code for training and evaluating the models used in this work is available at https://github.com/Garyqwt/Continuous_TFO.
