# Supplementary material for: Non-invasive detection of instantaneous fetal hypoxemia in large animal model of pregnancy
Source: NPJ Biomed Innov. 2025 Apr 23;2:12. doi: 10.1038/s44385-025-00014-0 (PMC12018256; doi:10.1038/s44385-025-00014-0)
Supplement: Supplementary file 1 — Supplementary Information [file 44385_2025_14_MOESM1_ESM.pdf]

10 Supplementary Material

10.1 Supplementary Notes: Details of Pregnant Ewe with In-Utero Hypoxic Fetal Lamb Model

To evaluate the novel Transabdominal Fetal Pulse Oximetry (TFO) system, a pregnant ewe model with an in-utero fetal lamb was used to conduct induced fetal hypoxemia experiments. The purpose of these experiments was to assess the performance of the TFO system against gold-standard fetal oxygenation measurements using ABG across varying levels of hypoxemia. This study was conducted under the approval of the Institutional Animal Care and Use Committee (IACUC) protocol 22476 at the University of California, Davis.

Each experiment involved a time-mated Dorper ewe of at least 132 days of gestation, with fasting for 18–24 hours before surgery (water was provided ad libitum). The gestational age for the five ewes used in this study were 138, 132, 141, 134, and 142 days.

At the time of surgery, anesthesia was induced using one of the following dosing regimens, depending on drug availability: a) 0.25mg/kg Diazepam plus 4–10mg/kg Ketamine; b) 2–6mg/kg Propofol plus 4–5mg/kg Ketamine; c) 0.05–0.5mg/kg Midazolam plus 4–10mg/kg Ketamine. Anesthesia was maintained with 1–4% isoflurane via inhalation, and the maternal respiration during the experiment was controlled by external ventilator.

To control the distal Mean Arterial Pressure (dMAP), an endovascular balloon catheter was placed through the sheath, into the femoral artery and advanced to the abdominal aorta. Placement was confirmed with x-ray or fluoroscopy, and blood pressure measurements were taken from pressure ports integrated into the catheter, above and below the balloon.

The sheep then underwent laparotomy and hysterotomy. After entering the uterine horn, the head of the fetal lamb was exposed, and an arterial line was placed in the fetal carotid artery, enabling hemodynamic monitoring and intermittent fetal blood sampling for Arterial Blood Gas (ABG) analysis. The fetal lamb was then returned to the uterus, and its ear was sutured to the uterine wall to secure its position. Amniotic fluid lost during the procedure was replaced with warm saline before the uterus and maternal abdomen were closed.

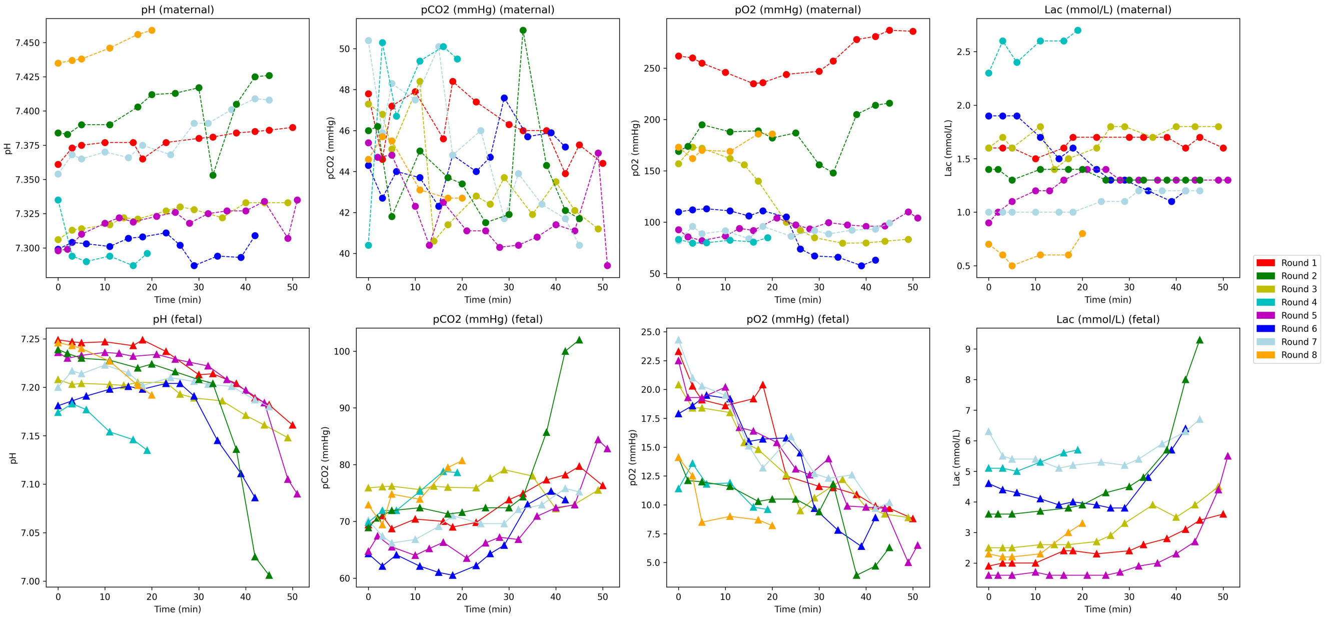

Supplementary Figure 1: Four additional ABG analysis are shown here including pH, pCO_2(mmHg), pO_2(mmHg), Lactate(mmol/L) for maternal and fetal measurements.

Subsequently, the TFO probe was placed on the maternal abdomen, positioning the LEDs directly above the fetal head. The endovascular balloon catheter was connected to a controllable manual syringe pump to adjust the balloon volume in real time, aiming to regulate dMAP, which in turn impacted placental MAP. Each hypoxic round followed a series of hypoxic steps, maintaining maternal dMAP at fixed levels. The steps began at 50mmHg dMAP, decreasing in 5mmHg steps via balloon inflation. If the baseline dMAP was below 50mmHg, the first step started at the nearest multiple of 5 below the baseline. Each hypoxic step lasted 10 minutes, with fetal blood samples collected at 2.5, 5, and 10 minutes for ABG analysis. The hypoxic steps concluded when two consecutive fetal arterial oxygen saturation ($fSaO_2$) readings were below 15%. After completion of a hypoxic round, the balloon was deflated, and a 45-minute recovery period followed. A new round was initiated if the fetal

$fSaO_2$ recovered to above 15%. Each sheep underwent a maximum of three rounds. ABG results from the eight hypoxic rounds used in this study are shown in Supplementary Figure 1.

At the end of the study, the fetus was euthanized via intravenous injection (IV) of 100mg/kg sodium pentobarbital or saturated KCl solution through the carotid catheter. The ewe was euthanized immediately after the experiment, by an IV injection of 100mg/kg sodium pentobarbital or saturated KCl solution, administered under general anesthesia.

The ARRIVE Essential 10: Compliance Questionnaire

Use this questionnaire to evaluate how well a manuscript complies with the ARRIVE Essential 10. It can be applied to any manuscript describing comparative experiments in living animals, by assessors such as journal staff, editors, or peer reviewers.

Item	Question(s)	Answers
1 Study Design	Are all experimental and control groups clearly identified?	☒ Yes, for at least one experiment<br>☐ No
	Is the experimental unit (e.g. an animal, litter or cage of animals) clearly identified?	☒ Yes, for at least one experiment<br>☐ No
2 Sample Size	Is the exact number of experimental units in each group at the start of the study provided (e.g. in the format '*n*=')?	☒ Yes, for at least one experiment<br>☐ No
	Is the method by which the sample size was chosen explained?	☒ Yes, for at least one experiment<br>☐ No
3 Inclusion & Exclusion Criteria	Are the criteria used for including and excluding animals, experimental units, or data points provided?	☐ Yes, for at least one experiment<br>☐ No
	Are any exclusions of animals, experimental units, or data points reported, or is there a statement indicating that there were no exclusions?	☐ Yes, for at least one analysis<br>☐ No
4 Randomisation	Is the method by which experimental units were allocated to control and treatment groups described?	☐ Yes, for at least one experiment<br>☐ No
5 Blinding	Is it clear whether researchers were aware of, or blinded to, the group allocation at any stage of the experiment or data analysis?	☐ Yes, for at least one experiment<br>☐ No
6 Outcome Measures	For all experimental outcomes presented, are details provided of exactly what parameter was measured?	☒ Yes, for at least one experiment<br>☐ No
7 Statistical Methods	Is the statistical approach used to analyse each outcome detailed?	☒ Yes, for at least one analysis<br>☐ No
	Is there a description of any methods used to assess whether data met statistical assumptions?	☒ Yes, for at least one analysis<br>☐ No<br>☐ Not applicable
8 Experimental Animals	Are all species of animal used specified?	☒ Yes, for at least one experiment<br>☐ No
	Is the sex of the animals specified?	☒ Yes, for at least one experiment<br>☐ No<br>☐ Not applicable to species
	Is at least one of age, weight or developmental stage of the animals specified?	☒ Yes, for at least one experiment<br>☐ No
9 Experimental Procedures	Are both the timing and frequency with which procedures took place specified?	☒ Yes, for at least one experiment<br>☐ No
	Are details of acclimatisation periods to experimental locations provided?	☐ Yes, for at least one experiment<br>☐ No
10 Results	Are descriptive statistics for each experimental group provided, with a measure of variability (e.g. mean and SD, or median and range)?	☒ Yes, for at least one experiment<br>☐ No<br>☐ Not applicable to the type of data collected
	Is the effect size and confidence interval provided?	☒ Yes, for at least one experiment<br>☐ No<br>☐ Not applicable to the type of analysis used

Notes on questionnaire design

The ARRIVE guidelines are a useful resource for authors preparing manuscripts describing animal research, and also provide a framework to evaluate the transparency of those manuscripts. To assess reporting quality, numerous studies have in the past sought to operationalise reporting guidelines (including ARRIVE). Typically, this involves scoring a manuscript's degree of compliance with guideline items in a binary fashion (e.g. an item is either not reported or reported) [1-3], a graded fashion (e.g. not, partially, or completely reported) [4,5], or a combination of the two [6].

This questionnaire has been designed to be as concise and user-friendly as possible. The number of questions used to assess a manuscript's compliance has been kept to a minimum, and in most cases each question is designed to be answered in a binary fashion. Compliance with some Essential 10 sub-items is inherently impossible to judge in this way, instead requiring a subjective judgement on the level of detail provided. For this reason, not all sub-items are represented by a question in this questionnaire.

To facilitate binary answers, it has been necessary to identify the minimum information in a manuscript sufficient to comply with each question. The strengths of this approach include the relatively short length of the questionnaire (and the correspondingly low time burden of using it), and the avoidance of ambiguity that would arise from a graded answering system, in which an intermediate score (e.g. 'partially/insufficiently reported') could denote a number of distinct deficiencies in compliance with an item (e.g. either only part of the item was complied with, or only the reporting of some experiments in the manuscript complied with the item.)

Limitations of this approach centre on the necessity to identify the minimum information sufficient to comply with each question. In some cases, this has resulted in questions that require a guideline sub-item's criteria to have been fulfilled in the reporting of only one experiment in a manuscript. As a result, not all experiments in a manuscript may be described in a way that fulfils that criterion, despite the manuscript being considered to comply with the guidelines overall.

References

1. Hair *et al* (2020). *Res Integ Peer Rev*. doi: 10.1186/s41073-019-0069-3
2. Tihanyi *et al* (2019). *J Surg Res*. doi: 10.1016/j.jss.2018.10.038
3. Zhao *et al* (2020). *BMC Vet Res*. doi: 10.1186/s12917-020-02664-1
4. Han *et al* (2017). *Plos One*. doi: 10.1371/journal.pone.0183591
5. Chatzimanouil *et al* (2019). *J Am Soc Nephrol*. doi: 10.1681/ASN.2018050515
6. Leung *et al* (2018). *Plos One*. doi: 10.1371/journal.pone.0197882
